# Liraglutide, a TFEB-Mediated Autophagy Agonist, Promotes the Viability of Random-Pattern Skin Flaps

**DOI:** 10.1155/2021/6610603

**Published:** 2021-03-31

**Authors:** Xuwei Zhu, Xinli Hu, Junsheng Lou, Jiafeng Li, Yu Xu, Gaoxiang Yu, Chenyu Wu, Jian Ding, Weiyang Gao, Jian Xiao, Kailiang Zhou, Chang Jia

**Affiliations:** ^1^Department of Orthopaedics, The Second Affiliated Hospital and Yuying Children's Hospital of Wenzhou Medical University, Wenzhou 325027, China; ^2^Zhejiang Provincial Key Laboratory of Orthopaedics, Wenzhou 325027, China; ^3^Molecular Pharmacology Research Center, School of Pharmaceutical Science, Wenzhou Medical University, Wenzhou 325027, China; ^4^Pediatric Research Institute, The Second Affiliated Hospital and Yuying Children's Hospital of Wenzhou Medical University, Wenzhou 325027, China

## Abstract

Random skin flaps are commonly used in reconstruction surgery. However, distal necrosis of the skin flap remains a difficult problem in plastic surgery. Many studies have shown that activation of autophagy is an important means of maintaining cell homeostasis and can improve the survival rate of flaps. In the current study, we investigated whether liraglutide can promote the survival of random flaps by stimulating autophagy. Our results show that liraglutide can significantly improve flap viability, increase blood flow, and reduce tissue oedema. In addition, we demonstrated that liraglutide can stimulate angiogenesis and reduce pyroptosis and oxidative stress. Through immunohistochemistry analysis and Western blotting, we verified that liraglutide can enhance autophagy, while the 3-methylladenine- (3MA-) mediated inhibition of autophagy enhancement can significantly reduce the benefits of liraglutide described above. Mechanistically, we showed that the ability of liraglutide to enhance autophagy is mediated by the activation of transcription factor EB (TFEB) and its subsequent entry into the nucleus to activate autophagy genes, a phenomenon that may result from AMPK-MCOLN1-calcineurin signalling pathway activation. Taken together, our results show that liraglutide is an effective drug that can significantly improve the survival rate of random flaps by enhancing autophagy, inhibiting oxidative stress in tissues, reducing pyroptosis, and promoting angiogenesis, which may be due to the activation of TFEB via the AMPK-MCOLN1-calcineurin signalling pathway.

## 1. Introduction

Random skin flaps are a useful tool in plastic and reconstructive surgery to address various skin defects caused by trauma, tumour resection, and diabetic wounds [1–3]. However, owing to an inadequate blood supply, when the ratio of width to length of the flap exceeds 1 to 1.5-2, necrosis of the distal flap occurs [4]. Inhibiting flap necrosis and promoting flap survival have become a major focus of flap research in recent decades. Flap necrosis primarily occurs due to ischaemia-reperfusion injury (IRI) caused by insufficient blood supply to the distal flap and postischaemic reperfusion, which leads to the production of reactive oxygen species (ROS). Simultaneously, the strong local inflammatory response that occurs after the flap operation also stimulates cell damage and necrosis in the flap tissue [5–7]. Therefore, previous studies have focused on the identification of specific drugs to inhibit oxidative stress, cell death, and inflammatory responses that promote tissue survival [8, 9].

Pyroptosis is a newly identified type of inflammatory programmed cell death [10]. The most common form of pyroptosis involves NOD-like receptor family pyrin domain containing-3 (NLRP3) activation, which initiates the self-cleavage of pro-caspase-1 to form mature caspase-1, which mediates the cleavage of the pyroptosis-related protein gasdermin D (GSDMD) [11–13]. The active form of GSDMD comprises the N-terminal domain, which is capable of aggregating to form pores in the cell membrane that promote the release of intracellular inflammatory factors, which eventually leads to the amplification of a cascade of inflammatory responses that results in focal necrosis. There are many upstream signals in the NLRP3 inflammasome, especially reactive oxygen species (ROS), which can stimulate the NLRP3 inflammasome and exacerbate the inflammatory response [14, 15]. Recent studies have demonstrated the role of pyroptosis in maintaining vascular homeostasis and in cardiovascular disease, with NLRP3 inflammasome-induced pyroptosis playing a key role in vascular endothelial dysfunction [15, 16]. Necrosis of the distal flap is a comprehensive reaction of vascular homeostasis, IRI, oxidative stress, inflammation, and cell necrosis. Therefore, there may be an important relationship between flap necrosis and pyroptosis.

Autophagy is a highly conserved cellular process that involves the degradation of damaged and inactive organelles, proteins, and nucleic acid, providing the necessary materials for cell reconstruction and regeneration to enable cell recycling [17, 18]. Previous studies have shown that the disruption of early autophagy pathways leads to the accumulation of ubiquitinated proteins, increased ROS levels, and mitochondrial dysfunction, resulting in increased oxidative stress [19, 20]. The induction of autophagy in diabetic mice can protect islet cells from oxidative stress [21]. Furthermore, in liver injury models, autophagy contributes to maintaining endothelial phenotypes and protecting liver cells from oxidative stress [22]. In addition, autophagy was observed to prevent pyroptosis by inhibiting caspase-1/GSDMD activation, while in one study of Crohn's disease, enhanced autophagy was observed to inhibit pyroptosis by inhibiting NLRP3 activation [23, 24]. Therefore, it is important to identify drugs that can inhibit oxidative stress and pyroptosis by activating autophagy, thereby promoting flap survival.

Liraglutide is a glucagon-like peptide 1 receptor agonist that has been reported to function as a novel type of antidiabetic drug with many benefits, including cardiovascular and neuroprotective effects [25, 26]. Previous studies have shown that liraglutide inhibits cardiac apoptosis and oxidative stress levels by activating AMP-activated protein kinase (AMPK) in diabetic rat models [27]. Liraglutide can reduce lipid accumulation, inhibit NLRP3 inflammasome activation and pyroptosis, ameliorate mitochondrial dysfunction and reactive oxygen species production, and enhance the mitochondrial phagocytosis of liver cells [28]. Liraglutide is an autophagy enhancer that has been used in a variety of fields [29, 30]. In addition, in diabetic wound models, liraglutide has also been demonstrated to promote angiogenesis [31]. Despite the notable potential of liraglutide, its use in random flaps is still new and requires further study. It is unclear whether liraglutide has beneficial effects on flap survival after reconstruction and transplantation. Therefore, the goal of the present study was to determine whether liraglutide can improve the survival of random skin flaps by regulating autophagy, oxidative stress, and pyroptosis and to explore its mechanism of action.

## 2. Materials and Methods

### 2.1. Experimental Mice

C57BL/6 mice (20-30 g, male) from the Wenzhou Medical University Animal Center (authorization number SCXK (ZJ) 2015-0001) were nurtured under normal conditions (humidity: 50 ± 5%; temperature: 23 ± 2°C) on a light/dark cycle for 12 hours, and the mice were allowed to eat and drink freely. Surgery, treatment, and perioperative care strictly adhered to the “Guide to the Care and Use of Laboratory Animals of the Chinese Institute of Health.” All operations involving mice were approved by the Animal Research Committee of Wenzhou Medical University (wydw2017-0022).

### 2.2. Antibodies and Reagents

The primary antibodies and reagents used in the present study and the companies from which they were purchased are as follows: Biogot Technology (Shanghai, China)—primary antibody against GAPDH; Boster Biological Technology, Ltd. (Wuhan, China)—primary antibody of cadherin 5; Beijing Solarbio Science & Technology Co., Ltd.—diaminobenzidine (DAB), pentobarbital sodium, and an H&E staining kit; Proteintech Group Inc. (Chicago, IL, USA)—primary antibodies against vascular endothelial growth factor (VEGF), matrix metalloproteinase 9 (MMP9), heme oxygenase 1 (HO1), (superoxide dismutase 1 (SOD1), cathepsin D (CTSD), vacuolar protein sorting 34 (VPS34), histone-H3, and caspase-1; Abcam (Cambridge, UK)—TRPML1/MG-2, primary antibodies against calcineurin, SQSTM1/p62, CD34 and ASC, and the secondary antibodies goat anti-rabbit IgG H&L (Alexa Fluor® 488) and goat anti-mouse IgG H&L (Alexa Fluor® 647); Cell Signalling Technology (Beverly, MA, USA)—primary antibodies against (endothelial nitric oxide synthase (eNOS), ATG-5, Beclin-1, NLRP3, mTOR, p-mTOR (ser2448), p-AMPK (ser485), and AMPK; Sigma-Aldrich Chemical Company (Milwaukee, WI, USA)—microtubule-associated proteins 1A/1B light chain 3 (LC3B) and 3-methyladenine (3MA); MedChemExpress (NJ, USA)—dorsomorphin (compound C); Bethyl Laboratories (Montgomery, TX, USA)—primary antibody against TFEB; ABclonal Technology (Wuhan, China)—primary antibodies against IL-1*β* and IL-18; Affinity Biosciences (OH, USA)—primary antibodies against GSDMD-N and p-TFEB (ser221); Shanghai Selleck Chemicals Co., Ltd. (Shanghai, China)—liraglutide (GLP-1 receptor); PerkinElmer Life Sciences (Waltham, MA, USA)—ECL Plus reagent kit; Beyotime Institute of Biotechnology (Jiangsu, China)—diamidino-2-phenylindole (DAPI) solution; Santa Cruz Biotechnology, Inc. (Dallas, TX, USA)—4,6-horseradish peroxidase- (HRP-) conjugated IgG secondary antibody; Thermo Fisher Scientific (Rockford, IL, USA)—NE-PER nuclear, cytoplasmic extraction reagents, and Pierce co-immunoprecipitation and BCA kits.

### 2.3. Random-Pattern Skin Flap Model

Mice were anaesthetized by an intraperitoneal injection of 1% pentobarbital sodium (*w*/*v*) at 50 mg/kg. After successful anaesthesia, a random skin flap (1.5 × 4.5 cm in size) was designed on the back of the mouse to cut the subcutaneous skin, and the subcutaneous trophoblast arteries at both ends of the pedicle of the flap were completely cut off. Finally, the isolated skin flap was sutured in situ with a 4-0 Johnson mousse thread. The random flap area was divided into the proximal end (area I), the middle (area II), and the distal end (area III), with each area being equal in size. In our previous studies, our flap model results showed that area III is typically completely necrotic, area I is completely alive, and area II exhibits an intermediate phenotype of necrosis and survival that is more sensitive to targeted therapy. Therefore, we selected area II as our experimental area to evaluate the effects of relevant treatments on inhibiting ischaemia and promoting flaps as well as to conduct further molecular biological examinations of the tissues in area II. On the seventh day after the flap operation, we euthanized all the mice with an overdose of pentobarbital. Six mice in each group were then randomly selected to macroscopically evaluate the flap, including by laser Doppler blood flow imaging, evaluation of the flap survival area, and evaluation of flap tissue oedema. Then, 6 mice from each group were randomly selected to extract proteins from flap area II tissue for Western blot analysis. Finally, 6 mice were selected to extract flap area II tissue for sectioning, and then the tissue sections were used for H&E staining, immunohistochemistry (IHC), and immunofluorescence analyses.

### 2.4. Adenoassociated Virus (AAV) Vector Preparation

The adenoassociated virus transcription factor EB (AAV-TFEB) shRNA used in our present study was constructed and packaged by Shanghai Gene Chemical Company (Shanghai, China). The TFEB-activated protein kinase shRNA sequence was synthesized and cloned into the pAV-U6-shRNA-CMV-EGFP plasmid to generate pAV-U6-shRNA (TFEB)-CMV-EGFP. AAV9-U6-shRNA (TFEB)-CMV-EGFP was obtained by transfection of AAV9-U6-shRNA (TFEB)-CMV-EGFP into AAV-293 cells with pAV-U6-shRNA (TFEB)-CMV-EGFP, adenovirus auxiliary plasmid (AD auxiliary plasmid), and an AAV Rep/Cap expression plasmid. AAV9-U6-shRNA (scramble)-CMV-EGFP was prepared using a similar process. The viral particles were purified using the iodixanol gradient method. The titres of AAV9-U6-shRNA (TFEB)-CMV-EGFP and AAV9-U6-shRNA (scrambled)-CMV-EGFP were 1.243 × 10^12^ and 1.22 × 10^12^ genome copies per ml, respectively, as determined by quantitative PCR.

### 2.5. Animal Drug and AAV Vector Management

One hundred thirty-two mice were randomly divided into six groups: the liraglutide group (*n* = 30), a control group (*n* = 30), the liraglutide+3MA group (*n* = 24), the liraglutide+scramble group (AAV-scramble control, *n* = 18), the liraglutide+AAV-TFEB shRNA group (TFEB short hairpin RNA, *n* = 18), and the LIR+CC (compound C, an AMPK blocker) group (*n* = 12). For the LIR (liraglutide) group, 200 *μ*g/kg of liraglutide was subcutaneously injected into mice daily starting 12 days before surgery and continued until the animals were sacrificed. In the control group, an equal amount of physiological saline was administered in the same manner. For the LIR+3MA group, 15 mg/kg of 3MA was subcutaneously injected into mice daily 30 minutes after liraglutide administration. Two days before liraglutide administration, the LIR+TFEB shRNA and LIR+scramble groups were injected daily with 100 *μ*l of PBS or viral vectors (a total of 1 × 10^10^ packaged genome-sized particles) into the tail vein, after which the LIR+TFEB shRNA and LIR+scramble groups continued to receive liraglutide following the same protocol as described for the LIR peptide injection. In the LIR+CC group, CC (1.5 mg/kg) was intraperitoneally injected 30 minutes before each injection of liraglutide.

### 2.6. Flap Survival Assessment

Flap survival was assessed by high-quality imaging on the third and seventh postoperative days. The flap images were evaluated using ImageJ (1.49v, National Institutes of Health, USA) to determine the proportion of flap survival area using the following formula: proportion of flap survival area = surviving area/total area × 100%.

### 2.7. Tissue Oedema Measurement

On the 7th day after surgery, the flaps of six mice from every group were cut down and weighed to determine the “wet weight.” The flaps were then dehydrated in an autoclaved kettle at 50°C and stabilized for 2 days. Then, the flaps were weighed again to determine the “dry weight.” The oedema degree of each flap was defined as the percentage of moisture observed on the 7th day after the operation and was calculated using the following formula: oedema degree of the flaps = (wet weight − dry weight) ÷ wet weight × 100%.

### 2.8. Laser Doppler Blood Flow (LDBF) Imaging

The vascular conditions of the flap were visualized by LDBF. On the 7th day after the operation, the anaesthetized mice were placed in the prone position, and the flaps were scanned by laser Doppler flowmetry (LaserFlo BPM, Vasamedics Inc., St. Paul, Minnesota), which allows the perforated branches of the blood vessels under the flap to be observed. The LDBF image was quantified using moorLDI (version 6.1). Blood flow intensity was calculated as perfusion units (PUs), and scanning was repeated three times.

### 2.9. Haematoxylin and Eosin (H&E) Staining

On the seventh day after the operation, the animals were euthanized, and 6 1 cm × 1 cm tissue specimens of flap area II were collected. These samples were fixed with 4% paraformaldehyde, paraffin-embedded, and then transversely sectioned. Then, 1 *μ*m thick slices were fixed on poly-L-lysine-coated slides for H&E staining. Subsequently, the number of microvesssels were counted for each sample from six randomly selected areas in 3 random sections under an optical microscope (Olympus Corporation, Tokyo, Japan) at 200x magnification, after which the number of microvessels per unit area (mm^2^) was calculated to quantify the blood vessel density.

### 2.10. Immunohistochemistry (IHC)

The six paraffinized sections described in the abovementioned section used for H&E staining were dewaxed with xylene and rehydrated in a graded ethanol bath for immunohistochemical analysis. The sections were washed, blocked with 3% (*v*/*v*) hydrogen peroxide solution, and then the slices were then soaked in 10.2 mM sodium citrate buffer for antigen repair. After thorough cooling, the slices were sealed with 10% goat serum (diluted in PBS) for 10 minutes, after which they were incubated with anti-CD34 (1 : 100), anti-cadherin 5 (1 : 200), anti-VEGF (1 : 200), anti-CTSD (1 : 100), and anti-SOD1 (1 : 100) primary antibodies overnight at 4°C in a wet box. The sections were then incubated with HRP-conjugated secondary antibodies, stained with a DAB detection kit, and counterstained with haematoxylin. A DP2-TWAIN image acquisition system (Olympus Corporation, Tokyo, Japan) was used to image the stained flap tissue sections at 200x magnification. ImageJ (1.49v, National Institutes of Health, USA) was used to quantify the density of CD34-positive vessels and the integral absorbances of cadherin 5-, SOD1-, VEGF-, and CTSD-positive vessels. IHC analyses were performed on 3 slices for each sample, with 6 random fields assessed for each slice.

### 2.11. Immunofluorescence Staining

Similar to the methods described for IHC, the sections were dewaxed, rehydrated, washed, and then soaked in 10.2 mM sodium citrate buffer for antigen repair. The slices were then washed with PBS 3 times, after which serum was dripped onto the samples and they were sealed in a wet box for 30 minutes. Then, the tissue sections were incubated with primary antibodies against GSDMD-N (1 : 100), caspase-1 (1 : 200), TFEB (1 : 100), and LC3II (1 : 200) at 4°C overnight. Subsequently, the specimens were incubated with a secondary antibody at room temperature in the dark for 1 hour and then stained with DAPI. Three sections were randomly selected from each specimen, and the dermis of 6 regions was observed and evaluated under a fluorescence microscope (Olympus Corporation, Tokyo, Japan). The percentages of LC3II-, caspase-1-, and GSDMD-N-positive cells and TFEB translocation to the nucleus were calculated.

### 2.12. Western Blotting

After the mice were euthanized, 0.5 × 0.5 cm samples of flap area II (*n* = 6) in each group were collected and stored at -80°C for subsequent Western blot analysis. After the flap tissue was extracted with lysis buffer, the protein concentration was measured using the BCA assay. Subsequently, 60 *μ*g of protein from each sample was separated by 12% (*w*/*v*) gel electrophoresis and then transferred to a polyvinylidene fluoride membrane (Roche Applied Sciences, IN, USA). The membrane was blocked with 5% (*w*/*v*) skim milk at room temperature for 2 hours and then incubated with primary antibodies against the following proteins at 4°C overnight: GAPDH (1 : 1000), cadherin 5 (1 : 1000), MMP9 (1 : 1000), VEGF (1 : 1000), SOD1 (1 : 1000), eNOS (1 : 1000), HO1 (1 : 1000), caspase-1 (1 : 1000), NLRP3 (1 : 1000), IL-18 (1 : 1000), IL-1*β* (1 : 1000), ASC (1 : 1000), GSDMD-N (1 : 1000), VPS34 (1 : 1000), Beclin-1 (1 : 1000), LC3 (1 : 500), p62 (1 : 1000), CTSD (1 : 1000), histone-H3 (1 : 1000), p-mTOR (1 : 1000), mTOR (1 : 1000), p-AMPK (1 : 1000), AMPK (1 : 1000), TRPML1/MG-2 (1 : 1000), calcineurin (1 : 1000), p-TFEB (ser221) (1 : 1000), and TFEB (1 : 1000). Finally, the membrane was incubated with an HRP-conjugated IgG secondary antibody (1 : 5000) for 2 hours, after which the immunoreactive bands on the membrane were observed with an ECL Plus reagent kit. Image Lab 3.0 (Bio-Rad, Hercules, CA, USA) was used to quantify the band intensity.

### 2.13. Statistical Analysis

Statistical analysis was performed with IBM SPSS Statistics version 25 (SPSS Inc., Chicago, IL, USA). All data are presented as the means ± standard error. An independent sample *t*-test was used to compare the mean values of two groups. One-way ANOVA was used to evaluate three or more groups. *P* < 0.05 was considered to indicate a significant difference.

## 3. Results

### 3.1. Liraglutide Promotes Flap Survival and Alleviates Tissue Oedema

**Figure 1:**
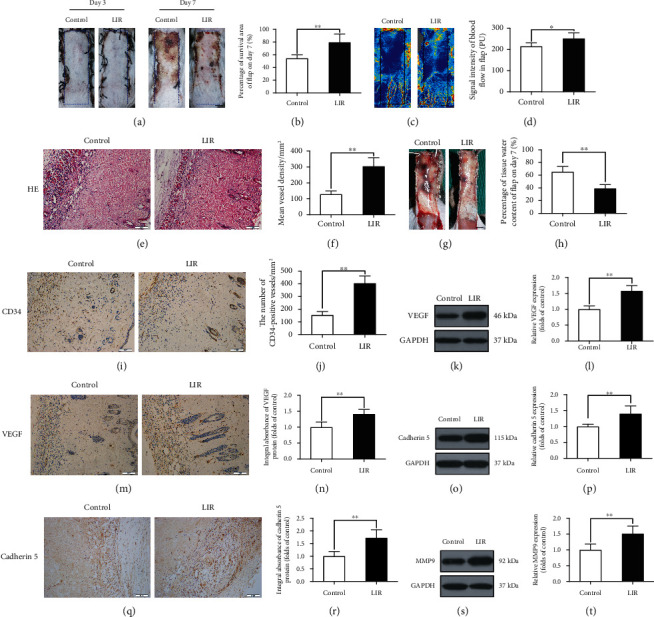
Liraglutide increases the survival rate of random flaps, reduces tissue oedema, and stimulates angiogenesis in flaps. (a) Images of the degree of flap necrosis in the control and LIR groups on the 3rd and 7th days after surgery (scale bar: 0.5 cm). (b) Histogram of the survival area percentage of the flaps on day 7 after surgery. (c) Laser Doppler blood flow scanning (scale bar: 0.5 cm) on the 7th day after surgery. (d) Histogram showing the intensity of the blood flow signal. (e) Haematoxylin and eosin (H&E) staining shows blood vessels in area II of skin flaps in each group (magnification: 200x; scale bar: 50 *μ*m). (f) Histogram of the mean vascular density (MVD) quantified from the H&E images. (g) Images of tissue oedema in each group on the 7th day after surgery (scale bar: 0.5 cm). (h) Histogram of the water content percentage of each tissue. (i, m, and q) Immunohistochemistry analysis of CD34-positive blood vessels and expression of vascular endothelial growth factor (VEGF) and cadherin 5 protein in flap area II for each group (magnification: 200x; scale bar: 50 *μ*m). (j, n, and r) Histogram of CD34-positive blood vessel density and the optical density values of VEGF and cadherin 5 from the immunohistochemistry results. (k, o, and s) Western blotting was used to detect the expression of VEGF, cadherin 5, and matrix metalloproteinase 9 (MMP9) in the control and LIR groups. (l, p, and t) ImageJ was used to quantitatively analyse the optical density values of VEGF, cadherin 5, and MMP9 in each flap group. The data are presented as the means ± standard error, *n* = 6 for each group. ^∗^*p* < 0.05 and ^∗∗^*p* < 0.01.

On the third day after surgery, skin flaps in each group were pale and exhibited oedemas, and there was no obvious necrosis in area III. There was no significant difference in the survival rate of the two groups (Figure 1(a)). On the seventh day after surgery, samples in both groups were still alive in area I, whereas area III (and part of area II) showed necrosis, blackening, and scabbling (Figure 1(a)). We observed that the flap survival rate of the LIR treatment group was significantly higher than that of the control group (Figure 1(b)). Specifically, the flaps were more swollen and damaged in the control group than in the LIR treatment group, and venous stasis was more obvious (Figure 1(g)). The water content of the flaps in the control group was significantly higher than that observed in the LIR group (Figure 1(h)).

Doppler blood flowmetry (LDBF) was used to evaluate the regeneration of the vascular network (Figure 1(c)), with the results showing that the blood flow signal was stronger in the LIR group than in the control group (Figure 1(d)). Vascular density was assessed by H&E histological staining, which showed that there were more microvessels in the LIR group than in the control group (Figure 1(e)). Quantitative analysis showed that the vascular density in the LIR group was higher than that observed in the control group (Figure 1(f)). Finally, endothelial cells in area II of the flaps were labelled with CD34 immunohistochemical analysis (Figure 1(i)). Compared to that observed in the control group, the number of CD34-positive vessels in the LIR group was significantly increased (Figure 1(j)).

### 3.2. Liraglutide Improves Random Skin Flap Angiogenesis

The survival of flaps depends on angiogenesis and an adequate blood supply. To investigate whether LIR contributes to angiogenesis in random flaps, we assessed the expression of neovascularization markers by Western blotting and immunohistochemistry (IHC) analysis. The Western blotting and IHC results revealed that VEGF expression in blood vessels and stromal cells in the LIR group was significantly increased compared to that observed in the control group (Figures 1(m), 1(n), 1(k), and 1(i)). In addition, compared to that observed in the control group, cadherin expression in stromal cells and blood vessels was also higher in the LIR group (Figures 1(q) and 1(r)). These findings were consistent with the Western blotting results (Figures 1(o) and 1(p)). MMP9 expression was also evaluated by Western blot (Figure 1(s)), suggesting that LIR regulates MMP9 expression (Figure 1(t)). Together, these findings suggest that LIR can promote VEGF, cadherin, and MMP9 expression, thereby promoting angiogenesis in flaps.

### 3.3. Liraglutide Alleviates Oxidative Stress in Flaps

**Figure 2:**
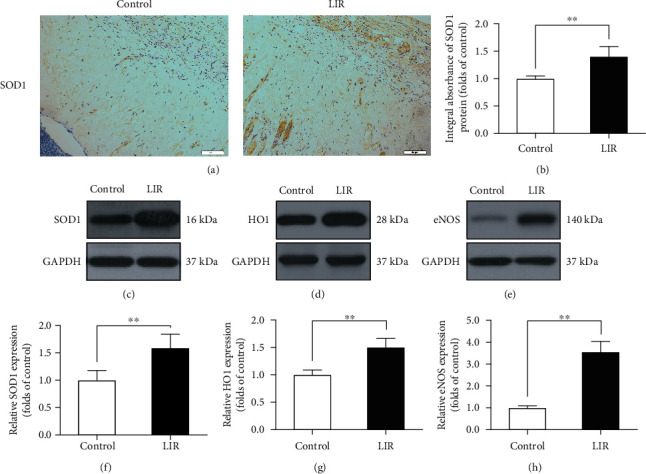
LIR reduces oxidative stress in random flaps. (a) Immunohistochemistry (IHC) was used to assess superoxygen dismutase 1 (SOD1) expression in each group (magnification: 200x; scale bar: 50 *μ*m). (b) Histogram of SOD1 absorbance. (c–e) The expression of SOD1, haem oxidase 1 (HO1), and endothelial nitric oxide synthase (eNOS) was determined by Western blotting. (f–h) ImageJ was used to quantitatively analyse the optical density of SOD1, HO1, and eNOS in each group. The data are presented as the means ± standard error, *n* = 6 for each group. ^∗^*p* < 0.05 and ^∗∗^*p* < 0.01.

Oxidative stress is an important factor leading to skin flap necrosis. To determine whether LIR can reduce oxidative stress, we used immunohistochemistry and Western blotting to detect the levels of proteins associated with oxidative stress. The results showed that SOD1 levels were higher in the LIR group than in the control group (Figures 2(a)–2(c) and 2(f)). Western blot analysis of HO1 and eNOS expression showed that the levels of these proteins were significantly increased in the LIR group (Figures 2(d), 2(e), 2(g), and 2(h)). These results showed that LIR decreased oxidative stress in flaps and significantly improved their activity.

### 3.4. Liraglutide Reduces Pyroptosis in Random-Pattern Flaps

**Figure 3:**
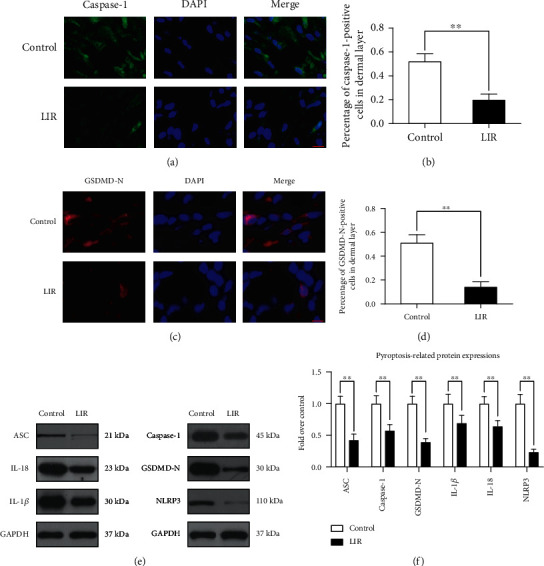
LIR inhibits pyroptosis in flap tissue. (a) Immunofluorescence (IF) assays were performed to evaluate caspase-1 levels in flaps from the control and LIR groups (scale bar: 10 *μ*m). (b) Histogram of the optical density value of caspase-1. (c) Immunofluorescence detection at the gasdermin D-N-terminal domain (GSDMD-N) level in the flap (scale bar: 10 *μ*m). (d) Histogram of GSDMD-N optical density values. (e) Western blot analysis of ASC, interleukin-18 (IL-18), interleukin-1*β* (IL-1*β*), caspase-1, GSDMD-N, and nucleotide binding oligomerization segment-like receptor family 3 (NLRP3) expression. (f) ImageJ was used to quantitatively analyse the optical density values of ASC, caspase-1, GSDMD-N, IL-1*β*, IL-18, and NLRP3 in each group. The data are presented as the means ± standard error, *n* = 6 for each group. ^∗^*p* < 0.05 and ^∗∗^*p* < 0.01.

Pyroptosis is an extremely important ischaemia-reperfusion injury. Immunofluorescence staining was performed to assess whether LIR could reduce the level of pyroptosis markers in flaps (Figures 3(a) and 3(c)). Quantitative analysis showed that LIR significantly decreased the percentage of caspase-1- and GSDMD-N-positive cells in the dermis of the flaps (Figures 3(b) and 3(d)). Western blot results showed that caspase-1, IL-18, IL-1*β*, ASC, NLRP3, and GSDMD-N levels were lower in the LIR group than in the control group (Figures 3(e) and 3(f)). Thus, LIR treatment appears to be capable of reducing pyroptosis levels in flaps.

### 3.5. Liraglutide Activates Autophagy in Random-Pattern Flaps

**Figure 4:**
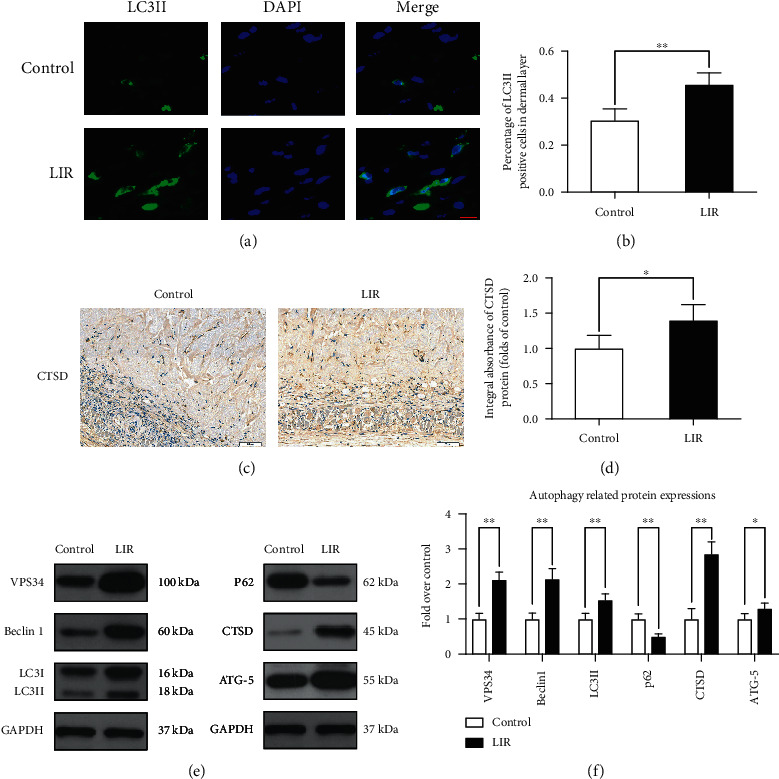
LIR activates autophagy in random flap tissue. (a) LC3II expression in flap tissues was detected by immunofluorescence analysis, and autophagosomes are shown (green) (scale bar: 10 *μ*m). (b) Histogram of the percent of LC3II-positive cells. (c) Immunohistochemical detection of CTSD expression in each group (magnification: 200x; scale bar: 50 *μ*m). (d) ImageJ was used to quantitatively analyse the CTSD optical density values for each group. (e) Western blot analysis of VPS34, Beclin-1, LC3II, P62, CTSD, and ATG-5. (f) ImageJ was used to quantitatively analyse the optical density values of VPS34, Beclin-1, LC3II, P62, CTSD, and ATG-5 in each group. The data are presented as the means ± standard error, *n* = 6 for each group. ^∗^*p* < 0.05 and ^∗∗^*p* < 0.01.

To assess the role of LIR in autophagy, we measured the levels of the major autophagy proteins in flaps, including CTSD (an autophagosome-related protein); LC3II, VPS34, and Beclin-1 (essential components of autophagosomes); and p62 (a substrate protein for autophagic flux). When LC3II-positive cells in the skin flaps were detected by immunofluorescence staining (Figure 4(a)) and their levels were compared to those observed in the control group, the number of LC3II-positive cells was significantly higher in the LIR group (Figure 4(b)). CTSD immunohistochemical staining results showed that CTSD levels were higher in the LIR group than in the control group (Figures 4(c) and 4(d)). In addition, Western blotting results showed that LC3II, Beclin-1, CTSD, and VPS34 levels were higher in the LIR group than in the control group. Correspondingly, p62 was expressed at low levels in the LIR group (Figures 4(e) and 4(f)).

### 3.6. Inhibition of Autophagy Reverses the Effects of Liraglutide on Oxidative Stress, Pyroptosis, and Angiogenesis and Reduces Random-Pattern Flap Survival

**Figure 5:**
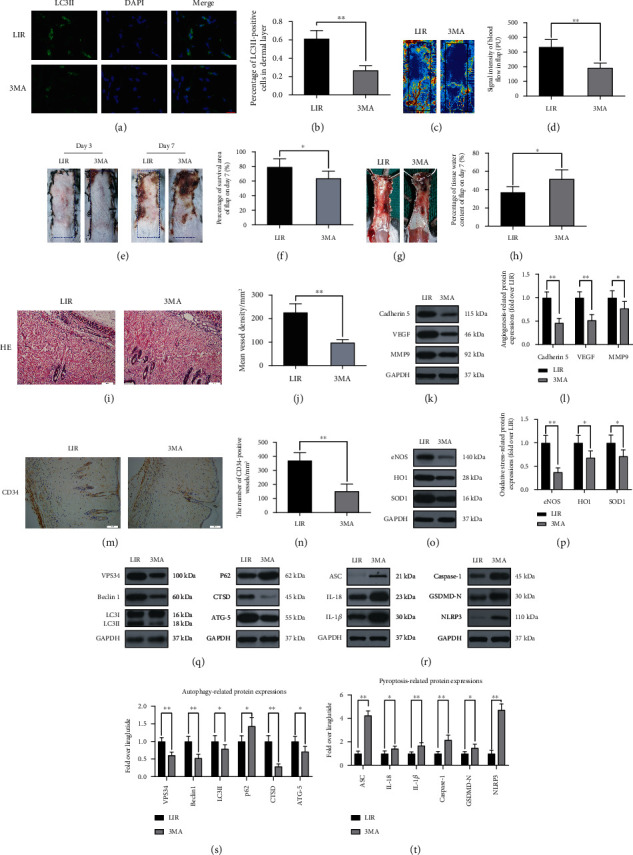
3MA reverses the effect of LIR in random flaps. (a) LC3II expression in flap tissues was detected by immunofluorescence analysis, and autophagosomes are shown (green) (scale bar: 10 *μ*m). (b) Histogram of LC3II-positive cell percentage. (c) Laser Doppler blood flow scanning (scale bar: 0.5 cm) on the 7th day after surgery. (d) Histogram showing the intensity of the blood flow signal. (e) The degree of flap necrosis in the LIR and LR+3MA groups on the 3rd and 7th days after surgery (scale bar: 0.5 cm). (f) Histogram of the survival area percentage of the flaps on day 7 after surgery. (g) Images of tissue oedema in each group on the 7th day after surgery (scale bar: 0.5 cm). (h) Histogram of the water content percentage of each tissue. (i) Haematoxylin and eosin (H&E) staining showing blood vessels in area II of skin flaps in each group (magnification: 200x; scale bar: 50 *μ*m). (j) A histogram of the mean vascular density (MVD) in H&E images was quantitatively analysed. (k and o) Western blots of cadherin 5, VEGF, MMP9, SOD1, HO1, and eNOS in the LIR and LIR+3MA groups. (i and p) ImageJ was used to quantitatively analyse the optical density values of cadherin 5, VEGF, MMP9, SOD1, HO1, and eNOS in each group. (m) Immunohistochemistry analysis of CD34-positive blood vessels in flap area II of each group (magnification 200x; scale, 50 *μ*m). (n) CD34-positive blood vessel density histograms. (q and r) Western blots were used to detect the autophagy indices for VPS34, Beclin-1, LC3II, P62, CTSD, and ATG-5 in each group and the pyroptosis indices ASC, caspase-1, GSDMD-N, IL-1 inhibitor, IL-18, and NLRP3. (s and t) ImageJ was used to quantitatively analyse the histogram of optical density values of autophagy and pyroptosis indicators in each group. The data are presented as the means ± standard error, *n* = 6 for each group. ns stands for nonsignificance, ^∗^*p* < 0.05 and ^∗∗^*p* < 0.01.

**Figure 6:**
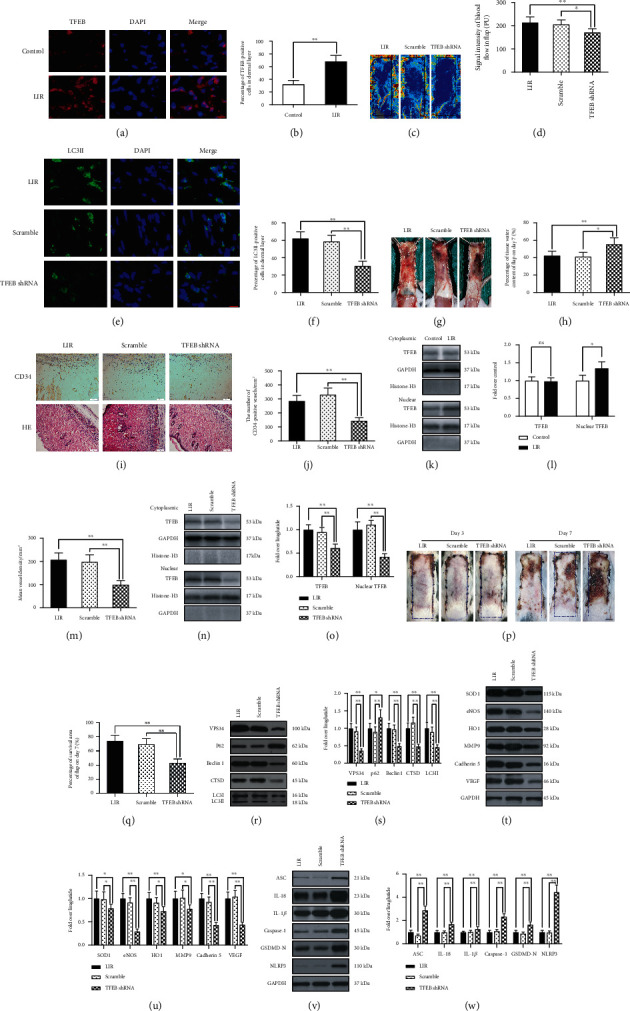
LIR promotes autophagy by activating TFEB activity. (a) On the 7th day after surgery, specimens were taken from the LIR, LIR+scramble, and LIR+TFEB shRNA groups for evaluation. Immunofluorescence analysis showed TFEB (red) nuclear translocation in the dermal cells of the flap (scale bar: 10 *μ*m). (b) Histogram of the TFEB-positive cell percentage. (c) Laser Doppler blood flow scanning (scale bar: 0.5 cm) on the 7th day after surgery. (d) Histogram of blood flow signal intensity. (e) Expression of LC3II in flap tissues was detected by immunofluorescence analysis, and autophagosomes are shown (green) (scale bar: 10 *μ*m). (f) Histogram of the LC3II-positive cell percentage. (g) Images of tissue oedema in each group on the 7th day after surgery (scale bar: 0.5 cm). (h) Histogram of the water content percentage of each tissue. (i) Immunohistochemistry analysis showing CD34-positive blood vessels in flap area II of each group, and haematoxylin and eosin (H&E) staining showing blood vessels in flap area II of each group (magnification 200x; scale bar: 50 *μ*m). (j and m) Quantitative analysis of CD34-positive vascular density and histograms of the mean vascular density (MVD) in H&E images. (k and n) Western blot analysis of cytoplasmic TFEB levels and nuclear TFEB expression. (l and o) Histogram of the optical density values of cytoplasmic TFEB nuclear TFEB in each group. (p) Images of the degree of flap necrosis in each group on the 3rd and 7th days after surgery (scale bar: 0.5 cm). (q) Histogram of the survival area percentage of skin flaps on day 7 after surgery. (r) Western blots of the autophagy-related proteins VPS34, p62, Beclin-1, CTSD, and LC3II. (s) ImageJ was used to quantitatively analyse the optical density of VPS34, p62, Beclin-1, CTSD, and LC3II in each group. (t) Western blots of the oxidative stress-related proteins SOD1, eNOS, and HO1 and the angiogenesis-related proteins MMP9, cadherin 5, and VEGF. (u) The optical density values of SOD1, eNOS, HO1, MMP9, cadherin 5, and VEGF in each flap. (v) Western blots of pyroptosis-related proteins ASC, IL-18, IL-1*β*, caspase-1, GSDMD-N, and NLRP3. (W) Quantitative analysis of the optical density values of ASC, IL-18, IL-1*β*, caspase-1, GSDMD-N, and NLRP3 in each group. The data are presented as the means ± standard error, *n* = 6 for each group. ^∗^*p* < 0.05 and ^∗∗^*p* < 0.01.

To determine whether the increased autophagy induced by LIR promotes flap survival, we used the autophagy inhibitor 3MA in conjunction with LIR and evaluated the resulting effects. First, Western blotting and IHC analysis were used to evaluate the levels of autophagy markers to verify that autophagy was indeed inhibited when 3MA was coadministered to flaps with LIR. Immunofluorescence staining results showed that the percentage of LC3II-positive cells in the flaps was significantly reduced in the LIR+3MA group (Figures 5(a) and 5(b)). In addition, LC3II, CTSD, VPS34, and Beclin-1 levels were significantly lower in the 3MA group than in the control group, while the levels of p62 were increased (Figures 5(q) and 5(s)), as observed by Western blotting. These results indicated that LIR-stimulated autophagy in random flaps was successfully inhibited by 3MA. Subsequently, we evaluated whether the combined administration of 3MA affected the efficacy of LIR in improving the viability of flaps. These results revealed that the rate of flap survival in the LIR+3MA group was significantly lower than that observed in the LIR group (Figures 5(e) and 5(f)). Similarly, 3MA aggravated the oedema and water contents of the flaps (Figures 5(g) and 5(h)). The LDBF results also revealed that 3MA decreased subcutaneous vascular density (Figure 5(c)), and the quantified difference was significant (Figure 5(d)). H&E staining results showed that the mean vascular density in the LIR+3MA group was significantly lower than that observed in the control group (Figures 5(i) and 5(j)), as was the number of CD34-positive vessels, as revealed through IHC staining (Figures 6(m) and 6(n)). In summary, these results suggest that 3MA significantly reverses the positive effect of LIR on flap survival, indicating that LIR improves flap survival by activating autophagy.

To further verify that autophagy is a major factor by which LIR promotes flap viability, we investigated the effects of 3MA on angiogenesis, oxidative stress, and pyroptosis. Compared to the LIR group, the expression of angiogenesis-related proteins (cadherin 5, VEGF, and MMP9) was significantly reduced in the LIR+3MA group, demonstrating that autophagy improved angiogenesis in the random-pattern flaps (Figures 5(k) and 5(l)). In addition, 3MA reduced the expression of oxidative stress protective proteins (eNOS, HO1, and SOD1) (Figures 5(o) and 5(p)). In contrast, the expression of pyroptosis proteins (IL-1*β*, IL-18, ASC, GSDMD-N, caspase-1, and NLRP3) was increased in the LIR+3MA group compared to that observed in the control group, indicating that autophagy inhibited the pyroptosis of flaps in the LIR treatment group (Figures 5(r) and 5(t)). In summary, our results showed that LIR increases autophagy in flaps, which is the primary mechanism by which LIR improves angiogenesis, reduces oxidative stress, inhibits cell pyroptosis, and ultimately improves the survival of flaps.

### 3.7. Liraglutide Activates Autophagy by Enhancing TFEB Activity

TFEB is an important regulator of autophagy. We observed that TFEB nuclear translocation in random flaps was significantly increased in the LIR treatment group compared to that in the control group (Figures 6(a) and 6(b)). Moreover, Western blotting results revealed a significant increase in nuclear TFEB level in the LIR treatment group (Figures 6(k) and 6(l)), but cytoplasmic TFEB level was nonsignificant in both groups. These results suggest that LIR promotes TFEB nuclear translocation, indicating that LIR enhances autophagy by activating TFEB. To further verify that TFEB activation enhances LIR autophagy, we used TFEB shRNA to silence TFEB activity and assayed the following groups: the LIR group, the LIR+scramble group (inactive shRNA), and the LIR+TFEB shRNA group. The results showed that the nuclear TFEB and cytoplasmic TFEB levels in the LIR+TFEB shRNA group were significantly lower than those observed in the LIR and LIR+scramble groups. No significant difference was observed between the LIR and the LIR+scramble groups (Figures 6(n) and 6(o)). These results indicate that TFEB shRNA successfully inhibited TFEB activation.

Subsequently, we investigated the effect of TFEB inhibition on LIR-induced flap autophagy. Immunofluorescence results showed no significant difference in the percentage of positive cells with LC3II-labelled autophagosomes between the LIR and LIR+scramble groups, while the percentage of cells testing LC3II-labelled positive cells in the LIR+TFEB shRNA group was significantly lower than that in the LIR+scramble group (Figures 6(e) and 6(f)). Similarly, Western blots showed no significant difference in VPS34, p62, Beclin-1, CTSD, and LC3II levels between the LIR and LIR+scramble groups, while the levels of LC3II, VPS34, CTSD, and Beclin-1 in the LIR+TFEB shRNA group were significantly lower than those observed in the other two groups, and the expression of p62 showed the opposite pattern (Figures 6(r) and 6(s)). Overall, these results reveal that TFEB dephosphorylation and nuclear translocation are the primary mechanisms by which LIR enhances autophagy.

Finally, we explored the effects of TFEB on angiogenesis, oxidative stress, and pyroptosis induced by LIR. TFEB shRNA treatment decreased the expression of angiogenesis-related markers (VEGF, MMP9, and cadherin 5) and oxidative stress markers (eNOS, HO1, and SOD1) and increased that of the pyroptosis markers (caspase-1, IL-18, IL-1*β*, ASC, NLRP3, and GSDMD-N). No significant difference in the expression of these proteins was observed between the LIR and LIR+scramble groups (Figures 6(t)–6(w)). The flap survival area of the LIR+TFEB shRNA group was significantly lower than those of the other two groups on the 7th day after surgery (Figures 6(p) and 6(q)). The TFEB shRNA treatment also caused more severe swelling and subcutaneous venous congestion, increased tissue moisture content of the flap, decreased subcutaneous blood flow signal intensity of the flap, and lowered vascular density compared to those observed in the other two groups (Figures 6(c), 6(d), 6(g)–6(j), and 6(m)). No significant difference in these variables was observed between the LIR+scramble and LIR groups (Figures 6(c), 6(d), 6(g)–6(j), and 6(m)). In summary, these results show that the positive effect of LIR on flap survival was mediated through TFEB nuclear translocation.

### 3.8. Liraglutide Activation of the AMPK-MCOLN1-Calcineurin-TFEB Signalling Pathway

**Figure 7:**
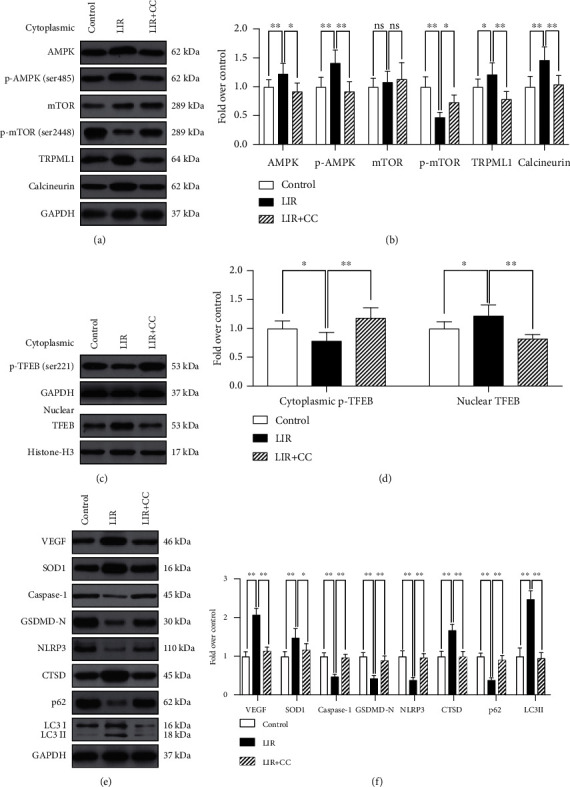
Liraglutide activates the AMPK-MCOLN1-calcineurin-TFEB signalling pathway. (a) On the 7th day after surgery, specimens were taken from the control, LIR, and LIR+CC groups for evaluation. Western blotting results showing AMPK, p-AMPK (ser485), mTOR, p-mTOR (ser2448), TRPML1 (MCOLN1), and calcineurin levels after internal GAPDH correction. (b) Histogram showing a quantitative comparison of AMPK, p-AMPK (ser485), mTOR, p-mTOR (ser2448), TRPML1 (MCOLN1), and calcineurin. (c) Western blotting results showing the level of cytoplasmic p-TFEB (ser221) after internal GAPDH correction, and the level of nuclear TFEB, which are corrected by histone H3. (d) Histogram showing the quantitative comparison of cytoplasmic p-TFEB (ser221) and nuclear TFEB levels between each group. (e) Western blot results revealing the protein levels of VEGF, SOD1, caspase-1, GSDMD-N, NLRP3, CTSD, p62, and LC3II corrected for GAPDH. (f) Histograms showing the quantified levels of VEGF, SOD1, caspase-1, GSDMD-N, NLRP3, CTSD, P62, and LC3II between each group. The data are presented as the means ± standard error, *n* = 6 for each group. ^∗^*p* < 0.05 and ^∗∗^*p* < 0.01.

TFEB activation is attributed to TFEB dephosphorylation, and the AMPK-mTOR1 signalling axis primarily functions by inhibiting TFEB phosphorylation, while another key pathway involved in this process is the calcineurin pathway. AMPK activation can promote the opening of MCOLN1 channels (also known as TRPML1 channels) in lysosomes and release large amounts of calcium ions. Calcium ions in the cytoplasm can activate calcineurin, which then dephosphorylates TEFB [32–34]. Therefore, we investigated whether the autophagy-promoting effect of liraglutide involves the AMPK-MCOLN1-calcineurin-TFEB signalling pathway. Based on our results, LIR increased p-AMPK (ser485) levels and reduced those of p-mTOR (ser2448), while no significant difference was observed in mTOR levels between the LIR and control groups (Figures 7(a) and 7(b)). The expression of the downstream signalling molecules MCOLN1 and calcineurin was also altered. As shown in Figures 7(a) and 7(b), the Western blotting results showed that the levels of MCOLN1 and calcineurin in the LIR group were significantly increased compared to those observed in the control group (Figures 7(a) and 7(b)). These results suggest that LIR activates the AMPK-MCOLN1-calcineurin pathway.

To investigate whether intracellular TFEB dephosphorylation after LIR treatment is regulated by the AMPK-MCOLN1-calcineurin signalling pathway, we used the AMPK inhibitor compound C to assess its effects on AMPK-MCOLN1-calcineurin signalling and flap survival. Our results suggest that AMPK-MCOLN1-calcineurin signalling pathway activation effectively increases TFEB dephosphorylation and nuclear translocation, which can be reversed by CC (Figures 7(c) and 7(d)). In addition, Western blotting results also showed that CC significantly inhibited the LIR-induced enhancement of autophagy and oxidative stress and the inhibition of pyroptosis in random flaps (Figures 7(e) and 7(f)). In summary, our results demonstrate that LIR enhances TFEB dephosphorylation in random flaps through the AMPK-MCOLN1-calcination signalling pathway.

## 4. Discussion

Random flap surgery is widely used, but flap necrosis is a common issue, and current treatment methods cannot completely solve the problem of distal flap necrosis. Thus, it is extremely important to identify novel drugs that can solve this issue. In the present study, we first discovered that liraglutide promotes angiogenesis, inhibits oxidative stress, and reduces pyroptosis in the ischaemic area by increasing autophagy, thereby promoting skin flap survival. Mechanistically, we first observed that liraglutide induces autophagy by activating the AMPK-MCOLN1-calcineurin-TFEB pathway and upregulating TFEB expression in flaps. These findings highlight the clinical potential of liraglutide to improve the prognosis of skin flap reconstruction surgery.

GLP-1 is an intestinal insulin-stimulating peptide that has several functions, such as lowering blood sugar and improving insulin resistance [35]. In recent years, liraglutide has received increasing attention for its angiogenic activity in addition to its traditional function of lowering blood glucose levels [36–38]. Liraglutide promotes angiogenesis to improve heart function [37], significantly increases hypoxia-inducible factor 1*α* (HIF1*α*) and VEGF expression during human umbilical vein endothelial cell (HUVEC) stimulation, and promotes angiogenesis [38]. Previous studies have shown that promoting angiogenesis is essential for the survival of flaps [5, 8, 39]. In our present study, treatment with liraglutide increased microvascular production in the random flap dermis, which improved the blood flow of the flaps and promoted their survival. Angiogenesis is a complex and tightly controlled process. VEGF specifically promotes the dilation, proliferation, and migration of endothelial cells, which is directly related to angiogenesis [40], with MMP9 being a major contributor to VEGF release and angiogenesis [41]. In addition, calcium adheres to death protein 5 to promote endothelial cell connections and prevent nascent blood vessel breakdown [42]. Our results show that VEGF, MMP9, and cadherin 5 protein levels significantly increased after liraglutide treatment, inducing angiogenesis, and promoting flap survival.

After reconstructive surgery, the far end of the flap will be in an ischaemic state, and as the blood supply recovers, the far end tissue may suffer I/R damage [43]. This situation may lead to the excess production of reactive oxygen species (ROS), which destroys the structure of cell membranes, nucleic acids, and chromosomes, triggering oxidative stress and accelerating cell death [44]. Antioxidant enzymes (e.g., SOD) are the primary cell defences against oxidative damage [45], with factors involved in antioxidant damage including haemoglobin oxygenase 1 and eNOS [46, 47]. Liraglutide was previously reported to improve oxidative stress in patients with T2DM [48], and another study confirmed that liraglutide also prevents vascular oxidative stress, thereby reducing cardiovascular complications associated with arterial hypertension [49]. Our results show that liraglutide increases the content of SOD1, eNOS, and HO1 in the flap dermis, proving that liraglutide suppresses oxidative stress.

Pyroptosis is a form of procedural cell death that causes rapid cell lysis [50] and is associated with a variety of physiological and pathological conditions, including oxidative stress damage caused by ROS overdose [51], which may lead to flap necrosis. Liraglutide has been reported to improve nonalcoholic fatty hepatitis and protect myocardial cells by inhibiting NLRP3 inflammasome-dependent pyroptosis [28, 52]. The NLRP3 inflammasome is a multiprotein platform that is activated when cells are infected or stressed, leading to the secretion of caspase-1-dependent proinflammatory cytokines (such as IL-1*β* and IL-18) that trigger pyroptosis [53, 54]. In addition, caspase-1 specifically cleaves between the amino ends of GSDMD and the cobs of the argon-based ends of the domain, which is essential for pyroptosis [53]. The results of our present study demonstrated that liraglutide can reduce NLRP3, ASC, caspase-1, IL-1*β*, IL-18, and GSDMD expression in the flap dermis, thereby inhibiting pyroptosis.

Previous studies have shown that autophagy plays a central role in promoting the viability of skin flaps [8, 9]. Autophagy is a conservative cellular process that maintains cell homeostasis by targeting lysosomal proteins and damaged organelles for lysosomal degradation [55]. Upon autophagy activation, the cell recycles damaged or excess cellular components by self-digestion, isolating them in a biomembrane component of the cytoplasm known as the autophagosome [56]. CTSD is an indicator of autophagosomes, and LC3II, VPS34, and Beclin-1 are markers of autophagosomes. In addition, p62 is a marker of autophagy substrate protein degradation [57, 58]. In our present study, we observed that LIR increased CTSD, LC3II, VPS34, and Beclin-1 expression while decreasing that of p62. We also used 3MA to inhibit autophagy to study the relationship between oxidative stress, pyroptosis, angiogenesis, and autophagy. After autophagy was inhibited, oxidative stress and pyroptosis were enhanced, angiogenesis was weakened, and flap activity continued to decrease. These results suggest that LIR inhibits oxidative stress and pyroptosis, increases angiogenesis by activating autophagy, and promotes flap survival. In summary, these results suggest that LIR promotes autophagy in random flaps.

To further elucidate the mechanism by which LIR promotes flap survival, we also explored the upstream mechanism of autophagy activity. Autophagy is a cellular catabolic process that is dependent on the coaction of autophagosomes and lysosomes. The transcription factor EB (TFEB) functions as the master regulator of lysosomal biogenesis by driving the expression of autophagy and lysosome genes [59–61]. In response to starvation or lysosomal dysfunction, cytoplasmic TFEB is dephosphorylated and translocates into the nucleus, where it positively regulates the expression of autophagy-associated genes (e.g., ATG-9, LC3, SQSTM1, and LAMP1) involved in a number of autophagy-related processes, from the identification of cargo and the formation of autophagosomes to vesicle fusion and substrate degradation [33, 59, 62]. In the present study, we observed that LIR increased TFEB expression and that autophagy activity was regulated by TFEB in the flap models. Taken together, our results suggest that LIR induces autophagy by promoting TFEB activation.

After showing that LIR induces autophagy by activating TFEB to promote random flap survival, we attempted to further elucidate how LIR regulates TFEB activity to provide a better foundation for its use in future clinical applications. Transcription factor EB (TFEB) colocalizes on the lysosomal membrane with the growth regulator mTOR. When nutrients are present, mTOR phosphorylation of TFEB inhibits TFEB activity. In contrast, during starvation or lysosomal destruction, mTOR activity is inhibited, disrupting TFEB phosphorylation and leading to its nuclear displacement [63, 64]. However, TFEB can only be phosphorylated in the nucleus. Previous studies have shown that lysosomal Ca^2+^ signal transduction controls the activity of calcineurin and its substrate, TFEB. Lysosomal Ca^2+^ activates calcineurin through the release of mucolipin 1 (MCOLN1, also known as TRPML1 channels), and TFEB binds to calcineurin to become dephosphorylated, thereby promoting its nuclear translocation [32, 33]. AMPK is essential for lysosomal function. AMPK inactivation inhibits lysosomal function by reducing the activity of the lysosomal calcium channel MCOLN1, while AMPK activation can reactivate MCOLN1, normalize lysosomal function, and induce autophagy [34, 65]. Subsequently, AMPK activation can inhibit mTOR activity and activate TFEB. Our results suggest that in the random flap models, LIR activates TFEB and stimulates autophagy through the AMPK-MCOLN1-calcineurin pathway. After treatment with AMPK inhibitor CC, TFEB activity was significantly inhibited, eventually leading to the inhibition of LIR-mediated autophagy. Therefore, the results of our present study demonstrated that LIR can stimulate the nuclear translocation of TFEB through the AMPK-MCOLN1-calcineurin pathway, thereby activating autophagy in random flaps.

Our study had some limitations that should be addressed in future investigations. First, as a conventional dose of liraglutide was used in the present study, other doses should be evaluated in future studies to identify the optimal dose for the treatment of flap necrosis and maximize its clinical value. Second, adenovirus transfection and gene knockout technology is still being developed. In future experiments, we will use additional gene knockout mice to perform relevant mechanistic research.

**Figure 8:**
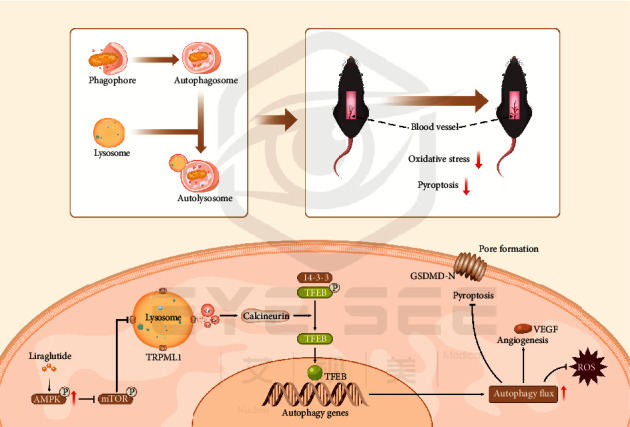
Mechanistic pathways by which liraglutide promotes flap survival through TFEB-mediated autophagy. Liraglutide activates the MCOLN1 channel (also known as TRPML1 channels) on lysosomes by stimulating AMPK, thereby releasing large amounts of Ca^2+^ and activating calcineurin. Calcineurin dephosphorylates TFEB, which then becomes activated and translocates into the nucleus, thereby stimulating autophagy. Upregulated autophagy inhibits oxidative stress and pyroptosis in flap tissues while stimulating angiogenesis.

In summary, the results of the present study provide new evidence that LIR activates TFEB through the AMPK-MCOLN1-calcineurin signalling pathway, leading to its dephosphorylation and nuclear translocation, thereby activating autophagy in random flap tissue. Increased autophagy can inhibit the pyroptosis of cells in random flaps, reduce oxidative stress, and promote angiogenesis, thereby increasing the survival rate of random flaps. A schematic illustration of our findings is presented in Figure 8. Our results provide strong evidence that LIR has good therapeutic benefits for random flaps, and we expect to translate LIR into the clinical use for random flaps as soon as possible.

## Data Availability

The datasets used and analysed during the current study are available from the corresponding authors on reasonable request.
